# Developmental outcomes with perinatal exposure (DOPE) to prescription opioids

**DOI:** 10.1515/nipt-2023-0017

**Published:** 2023-11-27

**Authors:** Adrian Flores, Nghi M. Nguyen, Gurudutt Pendyala

**Affiliations:** Department of Anesthesiology, University of Nebraska Medical Center (UNMC), Omaha, NE, USA; Department of Cellular and Integrative Physiology, UNMC, Omaha, NE, USA; Department of Genetics, Cell Biology and Anatomy, UNMC, Omaha, NE, USA; Child Health Research Institute, Omaha, NE, USA; National Strategic Research Institute, UNMC, Omaha, NE, USA

**Keywords:** perinatal, prescription opioids, epidemiology, preclinical, neurobehavior

## Abstract

Researchers have found considerable evidence in the past 20 years that perinatal opioid exposure leads to an increased risk of developmental disorders in offspring that persist into adulthood. The use of opioids to treat pain concerning pregnancy, delivery, and postpartum complications has been rising. As a result, communities have reported a 300–400 % increase in Neonatal Opioid Withdrawal Syndrome (NOWS). NOWS represents the initial stage of several behavioral, phenotypic, and synaptic deficits. This review article summarizes the Developmental Outcomes of Perinatal Exposure (DOPE) to prescription opioids. Moreover, we also seek to connect these findings to clinical research that describes DOPE at multiple stages of life. Since specific mechanisms that underlie DOPE remain unclear, this article aims to provide a framework for conceptualizing across all ages and highlight the implications they may have for longevity.

## Overview of opioids

Opioid receptors are a diverse class of receptors, consisting of the kappa opioid receptor (KOR), mu-opioid receptor (MOR), delta-opioid receptor (DOR), and Orphan Q. The distribution of these receptors is equally diverse, forming distinct niches across the body. Primarily located in the central nervous system and periphery [1], most of which are in the gut [2]. The function of the receptors is even more diverse, ranging from control of growth to excretion. However, this review will focus on those functions crucial to the general use and abuse of opioids. In the generalized signaling pathway pertinent to use and abuse, exogenous opioids depolarize neurons in the brain to lower the excitability of synapses responsible for pain sensation nociception [3, 4]. Pain relief is a crucial function to aid in survival following physical [5] and emotional trauma to some extent [6, 7]. Using this signaling pathway, scientists have developed agonists to help manage pain.

**Figure 1:**
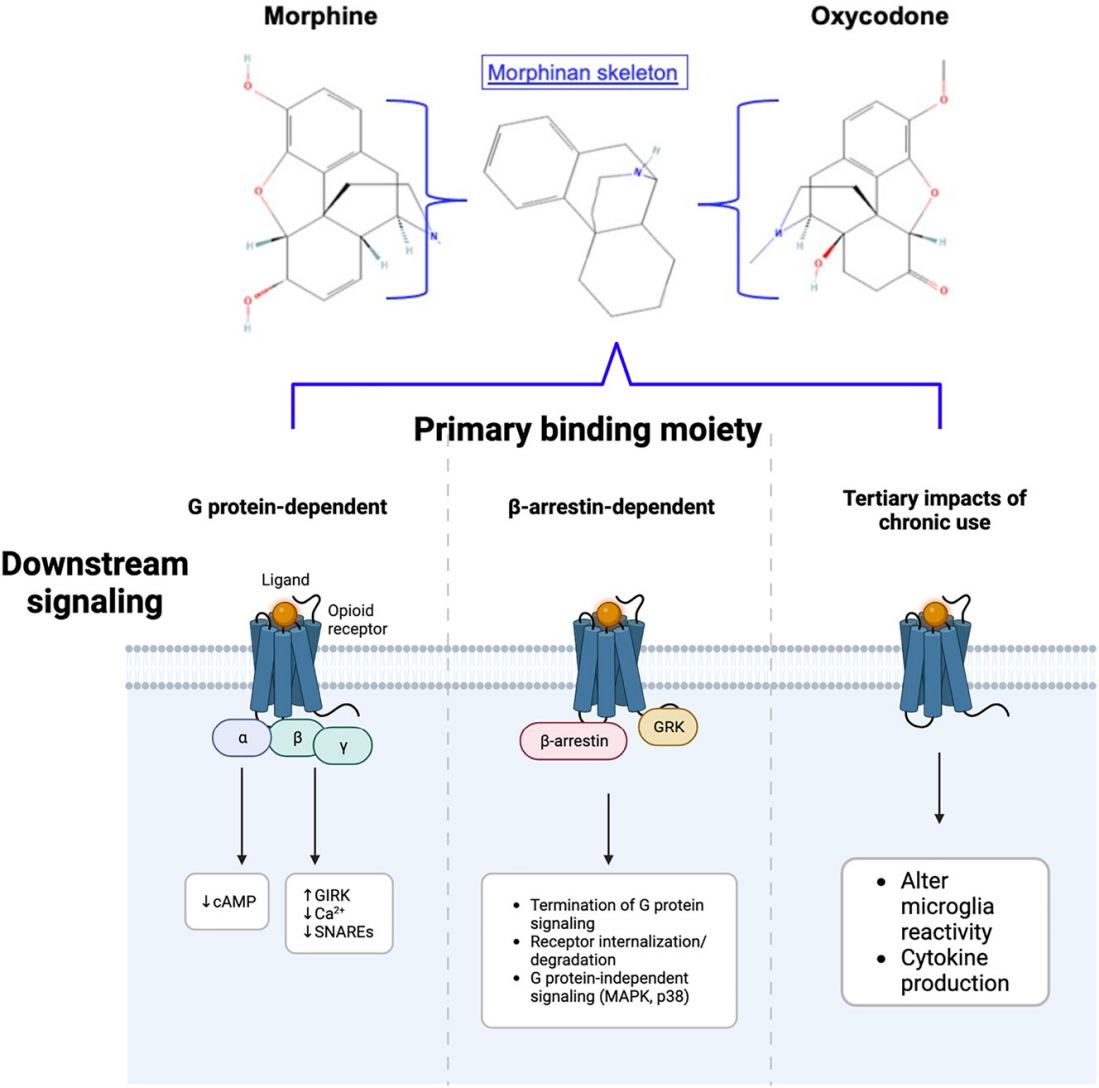
Common conserved binding element of opioids of focus, simplified downstream signaling mechanisms. Generated using biorender.com.

Prolonged use of opioids is known to lower the regulation of crucial neurotransmission in the limbic system. A summary of downstream signaling affected by opioids is shown in Figure 1. Opioid receptors are G Protein-Coupled Receptors (GPCRSs) with the potential to cause downstream alteration through β-arrestin signaling, such as phosphorylation of kinases, changes in NMDA receptor composition and activity, and purinergic receptor activity in microglia [2]. Presynaptic kinase disruption can lead to impairments in calcium signaling and changes in vesicle loading via changes in calmodulin and CAMKII [8]. Furthermore, opioid-dependent alteration to NMDA receptors is pivotal in maintaining ion concentration at the postsynaptic terminal [9]. These changes were found to contribute to excitatory changes in the limbic system that are a part of the addictive properties, possibly influencing the impulsivity of users [10].

Recently, scientists have expanded their understanding of the opioid receptor’s impact to include the immune system [11–14]. Acutely, opioids such as morphine are shown to blunt inflammatory responses to viral and bacterial infections, as shown in models using empty viral vectors and L.P.S. injections [15]. Researchers have found opioids such as morphine and oxycodone allosterically bind to Toll-like receptor 4 (TLR4) on T lymphocytes and B cells by interacting with myeloid differentiation factor 2 (MD2) [16]. MD2 is essential to differentiating T regulatory cells (T regs). Natural Killer cells (NK) are also impacted by opioid exposure, demonstrating a lowered ability to trigger apoptosis [17, 18]. More broadly than immunogenic properties of lymphocytes, opioid exposure has also been shown to alter the capabilities of surface-level receptors of peripheral blood mononuclear cells (PBMC)s [19, 20]. Collectively, research indicates that opioids impact the development of the immune system.

**Figure 2:**
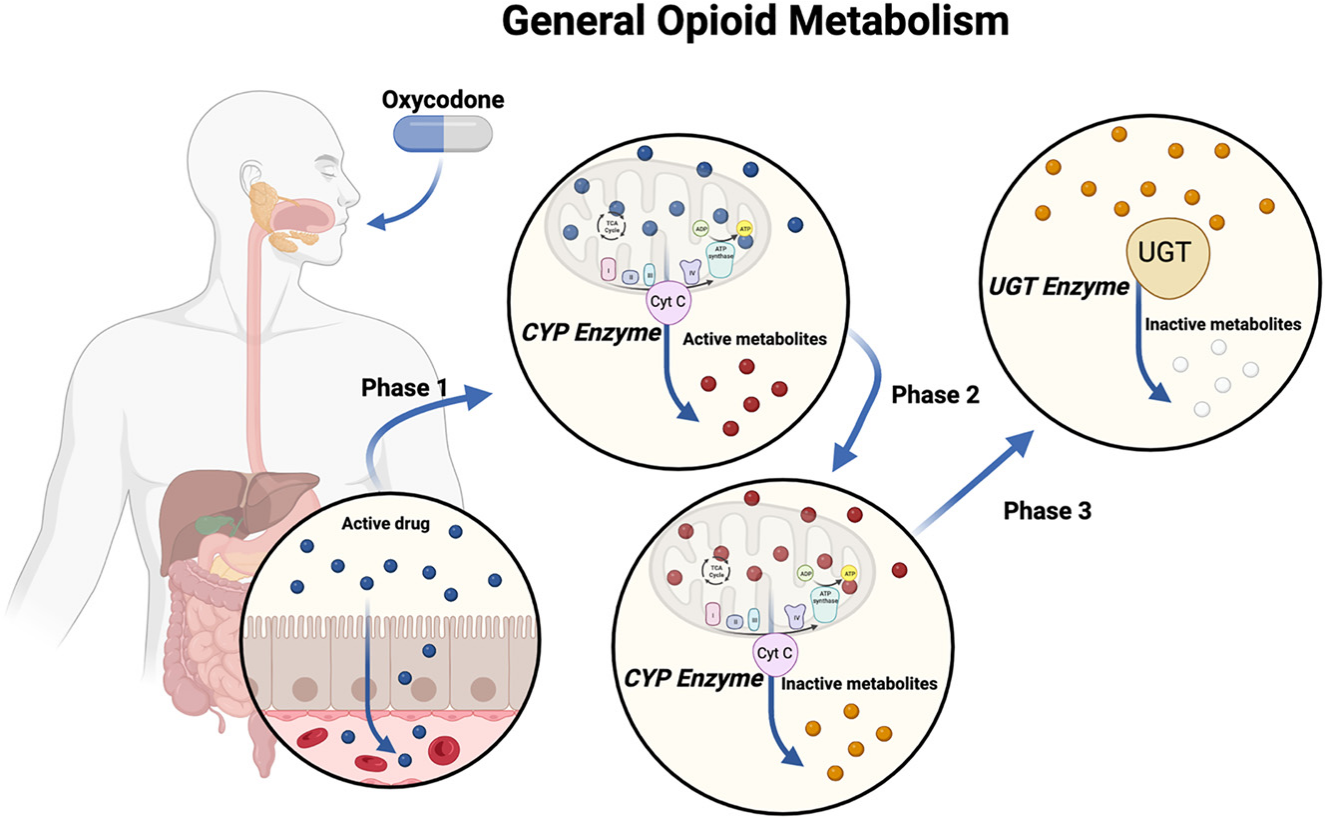
General opioid metabolism. Shown by example with oxycodone via oral administration. In the case of intravenous, phase one is completed more quickly, does not require absorption in the gut, and can directly interact with the liver, where all detoxification occurs. Generated using Biorender.com.

For the mechanism of action, this review will focus on the effects of MOR and KOR agonism. Drugs that target these pathways are commonly abused: heroin, morphine, hydrocodone, and oxycodone. The chemical structure of these drugs is highly conserved, sharing a morphinan ring fused to a piperdine ring, key to receptor agonism. Similarly, the metabolism of these opioids follows a common pathway. The opioid requires multiple passes with CYP enzymes in the liver before they can be glucuronidated and excreted. A simplified metabolic pathway for opioids is shown in Figure 2. From a pharmacodynamic and kinetic perspective, this allows adequate time for these drugs to work. Generally, CYP enzymes are highly inducible, producing significantly altered effectiveness in metabolizing other drugs (slower or more rapid metabolism). CYP induction has been shown with opioid metabolism in multiple instances [21]. This includes Gene Wide Association Studies (GWAS) showing an increased metabolism of opioids in regular users of opioids [22]. Additionally, opioids can be water or lipid-soluble, contributing to the rapid onset of each drug. In the case of our drugs of focus, commonly used analgesic opioids (morphine, buprenorphine, and oxycodone), they share a chemical structure that makes them lipophilic, allowing them rapid action and effective management of moderate and severe pain.

The latter niche (the gut) is crucial in balancing human metabolism – outside of directly inducing CYP enzymes. The gut also houses our microbiome, which plays a significant role in gastrointestinal health [2, 23]. Opioids reduce the excitability of nerves in the gut, leading to changes in motility, peristalsis, and intestinal permeability [23–25]. In recent years, scientists have become more aware of the delicate balance between gut and brain health, known as the gut-brain axis [26]. In addition to contributing to metabolism, the gut is a significant contributor of serotonin in the body, which directly communicates with the brain via the dorsal root ganglion. Researchers have found that chronic use of opioids leads to direct effects such as constipation but have also been shown to lower alpha diversity of microbiota [27], influencing gut-brain axis signaling. Scientists have also identified microbiota directly contributing to host immunity [28].

The metabolism and distribution of opioid receptors have predominantly been studied within the context of adult pharmacology and physiology. However, when examining the early stages of development, the complexity of opioid metabolism becomes more apparent. Researchers have utilized computational models to predict the fetal metabolism of opioids with varying degrees of success [29]. For example, computational predictions of fetal oxycodone and morphine metabolism have shown significant discrepancies when compared to clinical findings [30, 31]. Notably, these investigations have revealed distinct fetal deposition and metabolism patterns for morphine and oxycodone, despite both drugs sharing common metabolic pathways in adulthood. Furthermore, researchers have yet to fully characterize the derivates of fetal opioid metabolism. Together, this highlights a significant gap in our understanding of fetal opioid metabolism.

The opioid receptor’s role in early life is complex and changes based on the stage of development and tissue type. For example, trophoblast cells, dominate in early developmental tissue, release human chorionic gonadotropin (hCG) in response to KOR signaling [32]. However, this relationship shifts in later pregnancy when endogenous opioid signaling, mediated by molecules like endorphins, suppresses hormone release [33, 34]. At the villi, opioid signaling plays a significant role in blood flow [32]. Some research has indicated that exogenous administration of opioids can induce alterations in blood flow between the placenta and fetus, potentially leading to restrictions in nutrient and blood supply [31]. Some hypotheses suggest that this interaction may contribute to lower birth weight of perinatally exposed children. Additionally, research has demonstrated differences in the cellular localization of MOR and KOR in the brainstem in early life [35]. As mentioned earlier, opioids interact with TLR-4, which is immunologically active in the placenta, helping regulate immune responses [32]. In a rodent model Robertson et al., exogenous opioids have been shown to cause inflammatory changes in the placenta and fetus, possibly contributing to preterm birth [36]. In a separate rodent model, opioid receptors were significantly upregulated in the striatum in early life but were less functional than adult opioid receptors [37]. In summary, from a developmental perspective, it is clear that opioid signaling is a vital process that deviates from the functioning of opioid signaling in early development and adulthood.

### Opioid use disorder (OUD)

Opioids often induce sense of euphoria in many users. The euphoric effects of opioids have been well-documented throughout history, dating back to ancient civilization like the Greeks, often paired with a high potential for abuse and addiction. Notably, the effects of refined opiates, particularly opium, led to the addiction of millions in 19th-century China, triggering a national crisis. As mentioned earlier, opioid receptors are ubiquitous in mammalian physiology and are highly conserved.

In terms of addiction and abuse, opioids primarily work in the brain’s mesolimbic system, triggering hyperpolarization in this reward pathway. Unfortunately, the phenomenon of tolerance, which involves dose escalation, is common and rapid among opioid abusers. The danger lies in the downregulation of these receptors over time, which results in inadequate stimulation by endogenous production and necessitates dose escalation, eventually leading to the search for more potent receptor antagonists. Pharmacologists have sought to address this issue by developing more powerful opioids. As a result of the characteristics outlined here, opioids present significant physiological and psychological withdrawal symptoms.

Furthermore, tolerance and desensitization are only a few aspects of opioid use and abuse. Patients who do not manage their opioid doses correctly or engage in abuse often report hyperalgesia, experiencing significantly greater rebound pain compared to the ailment they were initially treating [38, 39]. Regarding opioid-induced hyperalgesia, research has consistently shown that pain thresholds are reduced in both patients and preclinical models [40, 41]. Additionally, opioids have a much broader long-term effect on neurotransmission.

In the era of modern pharmacology, synthetic opioids with potencies tens to hundreds of times greater than their historical counterparts have been developed. This paved the way for abuse of substances like heroin and morphine in the early 20th century. It is well-established that opioids have a high abuse rate and are highly addictive [17, 18]. Progress did not stop at morphine, as new drugs such as oxycodone, buprenorphine, and fentanyl have emerged, each carrying its own risk of future abuse. The abuse of these highly potent psychoactive analgesics has contributed to a significant loss of life through overdoses, neuronal hyperpolarization, and impacts on brainstem functions, including breathing control. To mitigate these overdoses, opioid receptor antagonists such as naloxone and naltrexone were developed.

In 1994, the American Psychological Association (A.P.A.) updated the DSM IV to formally recognize Opioid Use Disorder (OUD), as a distinct psychiatric disorder separate from other forms of substance abuse and dependency. Several therapeutics have been developed to facilitate a cessation of opioids and mitigate withdrawal symptoms, including medications like buprenorphine and methadone. Despite these advancements, the United States (U.S.) declared the Opioid Crisis a national public health emergency in 2018 [23, 27].

## The opioid epidemic

The use of opioids to treat chronic pain has seen a sharp increase since the late 1990s [42]. According to the Center for Disease Control (CDC) Wide-ranging Online Data for Epidemiological Research (WONDER), rates of OUD have surged by 500 % since the early 2000’s [42]. Anthropologists and sociologists attributed the start of the crisis to two main factors: (1) the misleading marketing of commonly prescribed opioids such as oxycodone to be non-habit forming [43] and (2) a campaign to make pain the “5th vital sign” [44]. Part of the campaign included marketing to physicians that when receptor desensitization and hyperalgesia occurred in patients, this was normal, and proper treatment involved doubling the opioid dose [45]. This directly contradicts anesthesiology research findings, which implied that to maintain effectiveness, dosage and pain should be closely monitored. While public awareness of the dangers of opioids has improved, recently Min et al. utilized the FDA’s Adverse Events Reporting System to show a significant rise in OUD and overdose using an interrupted time series analysis [46]. In the past three years, the U.S. has seen significant increases in the use of recreational fentanyl, leading to a spike in overdoses [47]. The continued abuse of opioids demonstrates the lasting impact of *The Opioid Epidemic* on American culture and public health.

Developmental biologists and neuroscientists recognize that the initial crisis and overdoses represent just the beginning of the problem. In the U.S., clinics are taking enhanced measures to monitor prescription opioids such as oxycodone [44, 48]. Hotspots of perinatal use reports are focused on rural communities and intercity areas [47, 49]. Areas with limited resources to track the affected individuals’ population suggest statistics on neonatal abstinence syndrome (NAS) incidence are conservative estimates. The next frontier will address the care and treatment of perinatally opioid-exposed children in the coming decades. To treat and care for these vulnerable populations, research must first accurately outline the developmental deficits of the offspring.

## The next national health crisis

The impacts of opioids extend beyond the individuals using them, with potential implications for the next generation. Perinatal opioid use presents a growing public health concern, particularly in terms of delayed maturation in perinatally opioid-exposed (POE) offspring. Over the last 30 years in the U.S., the use of prescription opioids to manage pregnancy-related breakthrough pain has steadily increased [42]. Prominent among these analgesics are opioids like buprenorphine, morphine, and oxycodone, which are crucial in various pregnancy contexts, including delivery, postoperative recovery, and managing persistent pains at home [50]. Opioid treatment is associated with the entire gestational cycle and even extending into lactation. Moreover, it is important to consider women who were using opioids to manage other symptoms before pregnancy and must continue usage throughout gestation in the actual clinical settings. It is well-established that all opioids can readily pass through the placental and fetal blood-brain barriers, accumulating in potentially harmful amounts [51]. Opioids also accumulate in biologically relevant concentrations in the breast milk of mammals during lactation [52–55]. Importantly, all opioids have the potential to impact offspring development. Even though some opioids, such as buprenorphine, have a lower risk of OUD and overdose than oxycodone and fentanyl. However, their impact on offspring development remains understudied, particularly within perinatal opioid exposure.

To compound this knowledge gap, we know less about the impact on immune cell development in the womb and their long-term impact on the offspring. This extends to the understanding of maternal immune activity and the development of mature lymphocytes and glial cells in the brain, which are physiologically relevant to establishing a healthy synaptic environment. Furthermore, a lack of research elucidates the direct impact of chronic and acute opioid exposure on placental and uterine tissue. While opioid receptors have been linked to the regulation of endometriosis [23], their definitive role in endometriosis and female fertility remains uncertain.

### Development theory

An overarching developmental theory posits that environmental, epigenetic, or pharmacological stressors during pregnancy can alter the development of offspring, resulting in a greater risk of disorders and diseases, a concept known as Baker’s theory of development. This theory is implicated in many perinatal stressors and the uptick in mental health challenges. Generally, the perinatal stressors affect the machinery involved in PANoptosis, a collective term for cell death processes including pyroptosis, apoptosis, and necroptosis. These disruptions can adversely affect the functioning of vital organ systems, including the brain [56, 57]. Such functional changes impact various aspects of physiology resulting global deficits. Specifically, several researchers have characterized deficits in immune signaling linked to gestational drug exposure (maternal immune deactivation) and maternal immune activation (M.I.A.) [58], which are associated with a greater risk of social disorders via synaptic deficits [59–62].

Opioids, including those used by pregnant women, have the potential to disrupt the immune profiles of users, as well as their offspring. In perinatal murine models of methadone exposure, researchers found distinct inflammatory profiles of exposed offspring that persisted into adulthood [51, 58, 63]. This indicates that immune impairments are not exclusive to the mothers taking opioids but extend to children exposed to them. Researchers hypothesize that inflammasome activity contributes to changes in the immune environment, potentially delaying synapse development [64]. In addition to these direct impairments, researchers have recently found POE to lead to microbiome dysbiosis in the dam and offspring [23, 27]. These foundational studies provide another mechanism to elucidate immune dysfunction in offspring and alterations in their baseline immune activity.

### The synapse development

Synactive theory of development is a branch of the overarching theory of developmental biology specific to synapse development [65, 66]. Generally, active synapses form more permeant neural circuits that stave off synapse elimination [67, 68]. Characterization includes a static timeline for neuronal, glial, and immune maturation [69]. The ability to eliminate and form synapses is also dependent on age [70, 71]. The density of potential neural connections peaks in early adolescence and stabilizes in late adulthood, with a decline as people reach the geriatric stages of life [72]. Synaptic plasticity follows a similar trajectory along with learning processes [73]. A hallmark of learning is making neural connections but also eliminating superfluous connections. Pruning of synapses is well illustrated in learning new languages, where eliminating erroneous sounds is just as crucial to speaking fluency [73]. A buprenorphine model of POE found offspring experience diminished learning capacity [74, 75]. This same trajectory of synapse formation is persistent among mammals such as primates and rodents. The elimination and formation of synapses are also controlled by the glial cells, such as astrocytes and microglia, through processes still under investigation.

### Role of glial cells

While scientists hypothesize that glial cells and activity are a driving force of synapse maintenance, there remains a disconnection between synapse formation and its overall functional neurodevelopment. For instance, Smith et al. demonstrated the pivotal physiological role of microglia in synapse pruning by depleting microglia in adolescent mice [64]. Similarly, perinatal TLR-stimulated offspring (similar to opioid stimulation of fetal TLR) were observed to have lower microglial counts and reduced complexity, coinciding with alternations in the electrophysiological properties of neurons [76]. Furthermore, Alipio and colleagues reported hyperactive basal synapse activity and evoked responses in POE offspring during adolescence, paired with changes in learning [52, 53]. Odegaard and colleagues also reported hyperactivity in the synapse, suggesting an impaired phenotype at the synaptic junction. However, works conducted by Wu and colleagues showed diminished synapse activity in the POE of buprenorphine, paired with increased depressive-like behaviors reported by reduced force swim test scores and lower reactivity of glial cells [74, 77]. In another study by Flores and colleagues, *in utero oxycodone exposed offspring* (IUO)-offspring displayed lower astrocyte markers in PFC-derived synaptosomes in an oxycodone model [78]. Taken together, the evidence suggests perinatal opioid exposure limits the glial cell’s ability to coordinate neuron growth and refining, leading to challenges in cognition.

Aside from potential developmental challenges, POE has the potential to be more susceptible to viral and bacterial infections. Newville and colleagues found that POE primes the peripheral immune system toward hyperactivity using an *ex vivo* approach [79]. Researchers have also seen significant evidence that microglia are active as viral reservoirs of pathogens such as HIV and COVID [80–82]. Specifically, buprenorphine is associated with increased infectivity of HIV in clinical evaluations of patient PBMCs [83]. As mentioned, PCR of brain tissue in POE offspring demonstrated a distinct phenotype with altered reactivity to nicotine, a typically neuroprotective substance [84]. However, research has yet to characterize the reactivity of these cells to viral or bacterial infection. This raises the question: are POE offspring’s microglia more amenable to high viral loads or more susceptible to HIV-associated neurocognitive disorders (HAND) or long-term COVID-19?

## Neonatal opioid withdrawal syndrome (NOWS)

Neonatal opioid withdrawal syndrome (NOWS) serves as a critical starting point for understanding the developmental outcomes with perinatal exposure (DOPE). This condition arises in children born to mothers who used opioids during pregnancy. This short window following delivery and feeding is enough to trigger withdrawal [51] in these offspring as they are accustomed to a continuous supply of opioids through the umbilical cord. The severity of withdrawal determines the level of care required for these children, placing a significant financial burden on the healthcare system. Taxpayers often shoulder this burden, as these children frequently receive care through Medicare resources. *In utero* models of opioid exposure provide insights into NOWS, as birth disrupts the continuous flow of opioids to the offspring, leading to considerable stress to the offspring during this short transitional period.

Notably, NOWS is a public health crisis that disproportionally impacts disenfranchised communities [85]. The rates of NOWS and NAS in the U.S. vary based on socioeconomic factors. For example, rural areas and minority populations report having 4–5 times NOWS cases. These disparities varied from access to treatment for use disorders and the level of maternal care routinely available to perinatal women. This includes the quality of communication between physicians and patients, which has been declining in the face of growing strains on healthcare systems and varies based on patients’ backgrounds [86].

Assessments for NOWS and neonatal abstinence syndrome (NAS) are in the early stages of development. Two prominent empirical methods for scoring the severity of NOWS and NAS are The Finnegan Neonatal Abstinence Scoring System (FNASS) and the Eat Sleep Console System (ESC) [87, 88]. These scoring systems have demonstrated their effectiveness in reducing the severity of infant stays in the Neonatal Intensive Care Unit (NICU) by providing crucial information to physicians about infant needs. However, these models of care are not standardized between NICUs. Thus, POE children often receive inadequate healthcare services within our current system.

While FNASS and ESC form an initial framework for pediatricians and psychiatrists to assess and treat infants, their utility is limited to the first few months. Importantly, the Diagnostic and Statistical Manual (DSM) includes OUD but lacks criteria for NAS and DOPE. This gap in behavioral sciences complicates tracking children’s development within the DOPE spectrum and underestimates affected populations. This paper seeks to bridge these gaps and advocate for DOPE’s inclusion in future DSM versions.

Clinical scientists have employed neuroimaging to demonstrate persistent differences in brain volumes of the frontal cortex, hippocampus, and amygdala definition in children with perinatal opioid exposure, which are critical for executive functions [89–91]. Simultaneously, researchers denote changes in limbic connectivity in children with POE through voxel-wise analyses [92]. Epidemiological data suggest an increased risk of psychiatric disorders, such as attention deficit disorder, in these children. Addressing the existing gap in clinical data is essential to comprehensively cover structural and connectivity changes in adolescents and adults exposed to opioids.

### General behavioral deficits

Beyond the cognitive implications, preclinical POE models have shed light on a range of behavioral deficits in offspring, including anxiety-like behaviors, depressive-like indicators, and analgesic deficits [93–96]. Among these, the most extensively studied deficit is anxiety-like behavior, which assessed through various assays such as open field, startle response, elevated plus maze, marble bury assay, and novel environment testing [97–102]. Persistent changes to analgesic tolerance were also observed in POE offspring, as indicated by hot plate, tail flick, and Von Frey assays. As mentioned earlier, POE offspring exhibit notably lower scores in forced swim tests, a decrease in social drive, and depressive-like indicators. Additionally, studies on vocalizations in basal and social testing settings have revealed altered stress tolerance and maternal interactions in POE offspring [103–106]. The implications of these findings extend beyond the individual and may involve factors such as maternal care or offspring brain connectivity.

Altered maternal care has been a concern among preclinical researchers, yet limited research has employed cross-fostering of POE offspring to disentangle direct causality in POE deficits. A search on PubMed and Google Scholar yielded only eight research articles addressing this limitation in perinatal research of analgesics, with just six articles focusing on opioids–none of which studied the impact of oxycodone [107–114]. Separate investigations focused on maternal care, outside of cross-fostering, found no significant difference in pup retrieval, which serves as a proxy for maternal care, between POE dams and control dams [96]. However, some other researchers found differences in vocalizations between dams, and the translational significance of these differences remains uncertain. These cross-fostering studies were further categorized into two prevailing influences on offspring well-being: (1) pre- and post-natal opioid exposure, and (2) maternal care. These studies provided a foundation for further investigation, noting differences between specific cross-fostering, including broad physiology and changes in the level of neurotransmitters.

Current research of pre- and post-natal opioid-exposed offspring in murine models suggests that these offspring are primed for unique developmental challenges. POE has been linked to behavioral deficits in stress tolerance at different stages of life, from early life to adulthood [52, 53, 115]. Furthermore, the timing of exposure has been shown to significantly influence the extent of deficits. For example, work performed by Smith and colleagues found robust sex-based differences in social behaviors among POE offspring exposed to morphine *in utero* [54]. Flores and colleagues have demonstrated that timing of exposure is crucial, with opioid exposure during the third trimester resulting in the most severe behavioral deficits in offspring compared to prenatal and continuous exposure [78].

It is also important to consider the challenges faced by children born needing surgery and treated with intravenous opioids for extended periods, commonly referred to as postnatally exposed individuals. These children often require more aggressive treatments, such as fentanyl, which has been associated with significant developmental deficits [15, 52, 53, 115]. Furthermore, these children may experience neonatal opioid withdrawal syndrome (NOWS), a topic of investigation by multiple researchers, revealing unique alterations in preclinical models [52, 53, 74, 93–95, 106, 115–118]. The growing epidemic of opioid use represents a pressing public health crisis, for which clinicians currently lack research-driven guidelines.

In addition to highlighting the significance of timing in offspring deficits, Flores et al. identified substantial evidence of sex-based alterations in body sizes and social behavior [78]. It’s worth noting that sex effects have been extensively documented in the field of drug abuse. Smith et al. also found sex-based behavioral differences in POE offspring. However, no studies have specifically focused on elucidating sex-based differences in both physiology and behavioral changes among POE offspring.

**Figure 3:**
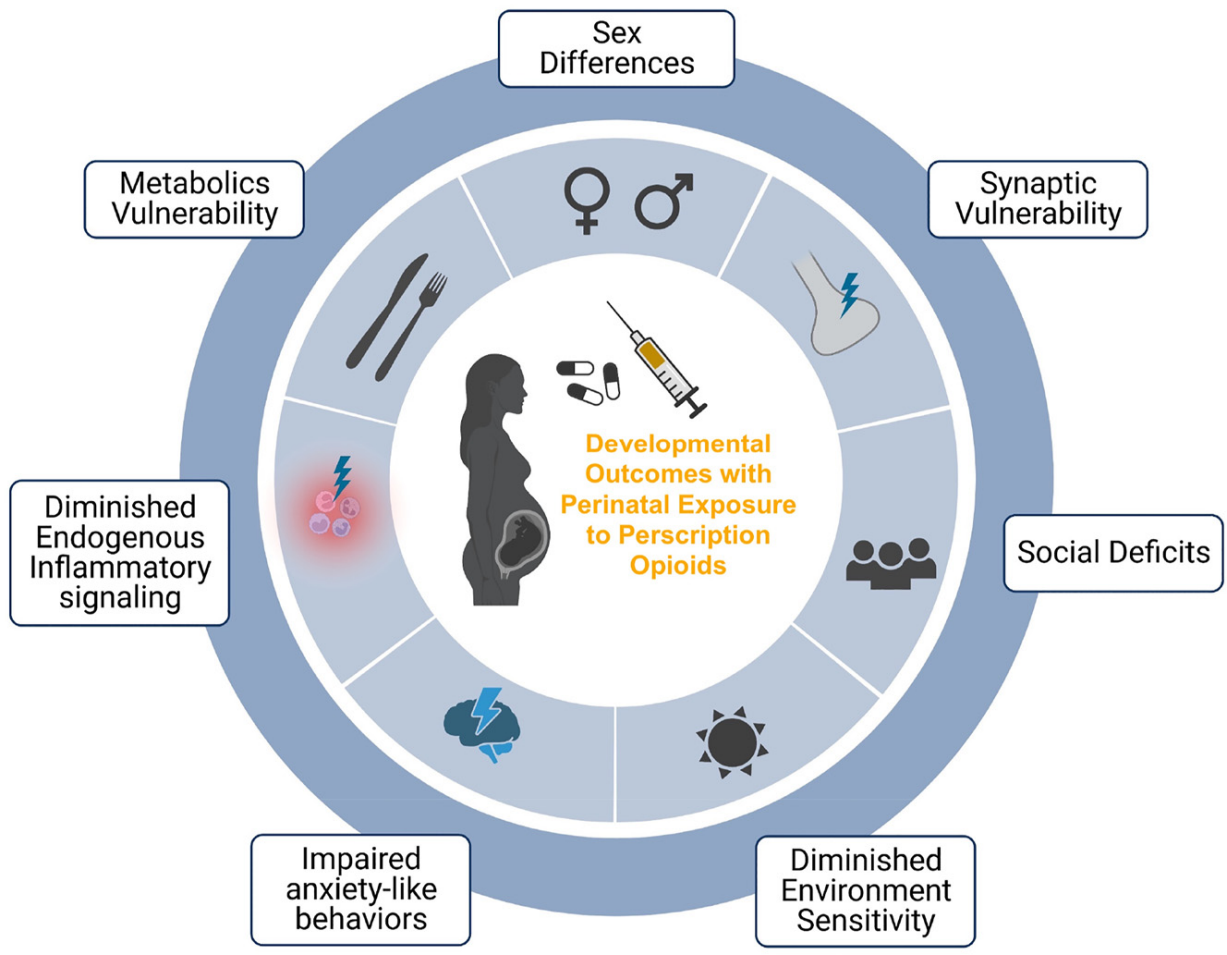
Overview of DOPE. Graphic summary of significant deficits found in using molecular, synaptic, and behavioral deficits observed in offspring. Generated using biorender.com.

Odegaard and colleagues have conducted comparative analyses of pre- and post-natally oxycodone-exposed offspring from early life to adulthood, highlighting the persistence of behavioral deficits [95]. Given the longer human gestational period compared to rodents, the impact of opioid exposure during the prenatal and early postnatal phases is more pronounced. Their chronic oxycodone model demonstrated significant and persistent anxiety-like effects in both pre-and postnatal oxycodone-exposed offspring, as indicated by behaviors such as marble burying during early life persisting into adulthood. These studies revealed that the severity of these impacts is influenced by the duration and timing of opioid exposure, with acutely exposed offspring experiencing more substantial and enduring deficits. Additionally, Flores and colleagues presented data from early adolescence using the same oxycodone exposure models, highlighting the significantly impaired sociability and responses to environmental stress compared to social stress among exposed offspring [78, 84]. A summary of DOPE impacts is shown in Figure 3.

## The perspective of future offspring investigation

One critical knowledge gap pertains to the cumulative impact of added life stresses on POE offspring. While recent research by Flores et al. has indicated that nicotine and other low-dose stimulants may not cause immediate harm to adolescent-exposed offspring, their vulnerability to other psychoactive substances like alcohol, anesthetics, and future analgesic use remains poorly understood [84]. These substances have been implicated in recent bioinformatics studies focused on the transcriptional and translational level [54, 78, 95, 96]. However, comprehensive investigations into the susceptibility of POE offspring to future substance use, using methods such as self-administration or preference testing are lacking. Notably, perinatal heroin exposure has been shown to induce enhanced self-administration in offspring, but the patterns of administration of commonly abused substances like prescription opioids, alcohol, and caffeine remain unknown.

Gowen and colleagues have recently also shown that POE offspring are more susceptible to mild traumatic brain injury (mTBI) using a robust rotational brain injury model in conjunction with prenatal opioid exposure [96]. This study also revealed that mTBI exacerbated injury-related changes in immune profiles and oxidative stress at the mitochondrial level, finding dysfunction in multiple complexes in the electron transport chain. However, this study primarily focused on mTBI induced in early life, and further work is needed to confirm the persistence of changes in offspring recovery profiles.

Another unexplored area relates to the consequences of intergenerational exposure on offspring. Although preliminary characterizations of F2 offspring behavior have been conducted by Odegaard and colleagues, no mechanistic validation for F2 deficits has been established [94]. These studies mainly emphasized maternal influences of opioid exposure, with a lack of male subjects to form the F2 generation through mating. The omission of male heritability as a mechanism for deficits is a notable limitation in all existing POE models.

Despite recent advancements in our knowledge regarding the early stages of POE offspring, significant gaps persist. Little is known regarding the geriatric pathology of these offspring and whether their longevity is affected in the long term. Specifically, it remains unclear whether these populations are more susceptible to degenerative neurological diseases like Alzheimer’s or other forms of dementia furthermore, while research on HIV/AIDS has garnered substantial funding, addiction studies, including POE research, have received considerably less support. In fact, addiction studies receive only a fraction of the funding allocated to HIV/AIDS research [119], resulting in a lack of investments in the study of significantly aged POE offspring.

## Conclusions

The Opioid Epidemic has sparked a new public health crisis, particularly concerning the care of perinatally opioid-exposed children. While progress has been made in characterizing baseline deficits, significant knowledge gaps persist. Adolescence, a period of high neuroplasticity and habit formation, remains poorly understood, leaving critical intersections unexplored. Moreover, comprehensive insights are lacking into the potential effects of future drug use, traumatic experiences, social stress, and brain injuries on these populations.
